# The Use of Intact Fish Skin as a Novel Treatment Method for Deep Dermal Burns Following Enzymatic Debridement: A Retrospective Case-Control Study

**DOI:** 10.3390/ebj3010006

**Published:** 2022-01-27

**Authors:** Christoph Wallner, Jana Holtermann, Marius Drysch, Sonja Schmidt, Felix Reinkemeier, Johannes Maximilian Wagner, Mehran Dadras, Alexander Sogorski, Khosrow Siamak Houschyar, Mustafa Becerikli, Marcus Lehnhardt, Björn Behr

**Affiliations:** Department of Plastic Surgery, BG University Hospital Bergmannsheil, Ruhr University Bochum, Bürkle-de-la-Camp Platz 1, 44789 Bochum, Germany; jana.holtermann@gmx.de (J.H.); marius.drysch@bergmannsheil.de (M.D.); Sonja.Schmidt@bergmannsheil.de (S.S.); Felix.Reinkemeier@bergmannsheil.de (F.R.); johannes.wagner@bergmannsheil.de (J.M.W.); Mehran.Dadras@bergmannsheil.de (M.D.); Alexander.Sogorski@bergmannsheil.de (A.S.); Khosrow-Houschyar@gmx.de (K.S.H.); Mustafa.Becerikli@bergmannsheil.de (M.B.); marcus.lehnhardt@bergmannsheil.de (M.L.); bjoern.behr@bergmannsheil.de (B.B.)

**Keywords:** severe burn injuries, acellular fish skin graft, wound matrix, xenotransplantation, wound healing, scarring

## Abstract

Background: The optimal therapy for deep burn wounds is based on the early debridement of necrotic tissue followed by wound coverage to avoid a systemic inflammatory response and optimize scar-free healing. The outcomes are affected by available resources and underlying patient factors, which represent challenges in burn care and suboptimal outcomes. In this study, we aimed to determine optimal burn-wound management using enzymatic debridement (NexoBrid™, MediWound Germany GmbH, Rüsselsheim, Germany) and intact fish skin (Kerecis^®^ Omega3 Wound, Isafjordur, Iceland). Methods: In this retrospective case series, 12 patients with superficial or deep dermal burn wounds were treated with enzymatic debridement followed by fish skin, Suprathel^®^ (PolyMedics Innovations GmbH, Denkendorf, Germany), or a split-thickness skin graft (STSG). Patients’ outcomes regarding healing and scar quality were collected objectively and subjectively for 12 months after the burn injury. Results: Wounds treated with fish skin demonstrated accelerated wound healing, a significantly higher water-storage capacity, and better pain relief. Furthermore, improved functional and cosmetic outcomes, such as elasticity, skin thickness, and pigmentation, were demonstrated. The pain and itch expressed as POSAS scores (Patient and Observer Scar Assessment Scale) for fish skin decreased compared to those for wounds managed with an STSG or Suprathel. Importantly, fish skin-treated wounds had significantly improved sebum production and skin elasticity than those treated with Suprathel but showed no significant superiority compared to STSG-treated wounds. Conclusions: Enzymatic debridement in combination with intact fish skin grafts resulted in the faster healing of burn wounds and better functional and aesthetic outcomes than split-thickness skin grafts and Suprathel treatment.

## 1. Background

Over the past 50 years, advances in burn care have led to a massive improvement in clinical care and a subsequent decrease in burn-patient mortality [1]. The systemic inflammatory response to burn wounds reduces muscle mass and impairs wound healing [2,3]. The principle of the early debridement of necrotic tissue followed by wound coverage has been established for extensive burns to counter this inflammatory response. The current standard of care for deep burns consists of early excision followed by split-thickness skin grafting (STSG). This approach reduces recovery time and has fewer complications, such as sepsis, multi-organ failure, and acute kidney injury [4,5].

Despite the advances in burn care, there are still limitations for optimal burn management. These include, in some cases, a lack of available infrastructure and resources, as well as challenges relating to patients’ underlying comorbidities [1,5]. With the development of enzymatic debridement (NexoBrid™, MediWound Germany GmbH, Rüsselsheim, Germany), a selective bedside procedure can be performed, allowing for the very early and less traumatic debridement of necrotic tissues [6,7,8]. Moreover, several studies have demonstrated a reduced time from injury to complete debridement, with decreased blood loss and excision, fewer surgical procedures, and a reduced need for autografting reported [8,9,10,11,12].

In particular, the coverage of large burn areas remains challenging. In these cases, autologous skin grafts are limited in availability; allogenic and xenogenic skin substitutes have gained traction and are commonly used for temporary coverage until donor sites can be reharvested for grafting or healing through secondary intention [1,13]. Skin substitutes from pigskin or human cadaver skin carry the risk of autoimmune responses and infections, and additional challenges include cultural and religious barriers, as well as the disruption of the skin’s architecture [4]. A novel skin substitute from acellular fish skin graft (Kerecis^®^ Omega3 Wound, Isafjordur, Iceland) has gained attention for the treatment of burn patients. The gentle processing of north Atlantic cod (*Gadus morhua*) skin allows it to maintain its nutrients, including omega-3 polyunsaturated fatty acids, providing potential anti-inflammatory and antimicrobial benefits. Fish skin has not been linked to autoimmune reactions in previous studies [14,15,16,17,18] and does not have any cultural or religious barriers to use. Its porous microstructure supports dermal cell migration, proliferation, and ingrowth [15,17]. Fish skin grafts have successfully treated complicated vascular and diabetic ulcers with exposed tendons and bones [17]. Increased wound-healing rates and rapid wound-surface-area reductions for full-thickness diabetic ulcers have been observed [18,19]. Studies and reports on burn patients treated with Kerecis Omega3 Wound are limited but suggest similar effects with promising outcomes [4,20].

The current study aimed to determine if a combined treatment with NexoBrid and fish skin graft was equal to or better than the current standard of care. Therefore, burn injuries were covered with fish skin graft, an STSG, or Suprathel^®^ (PolyMedics Innovations GmbH, Denkendorf, Germany), depending on the wound depth. As addressing scarring remains a challenging part of burn care, the focus was on the progression of wound healing and the scar quality of healed burn wounds for validation [1,21].

## 2. Methods

### 2.1. Selection of Subjects

**Figure 1:**
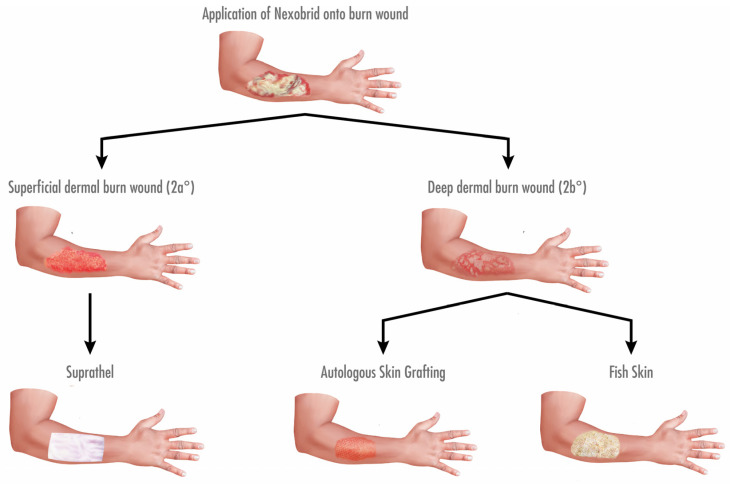
Algorithm for treatment of dermal burn wounds in our facility regarding debridement and coverage. Burn wounds were subjected to enzymatic debridement using NexoBrid on the second day after admission. Subsequently, wound depth was determined. Superficial dermal burn wounds (2a°) were covered with Suprathel, while deep dermal burn wounds (2b°) were treated with either an autologous STSG or fish skin graft.

Data from patients (age range: 18 to 60 years old) with mixed dermal burn wounds receiving subsequent treatment with at least two different wound-cover techniques, including Kerecis Omega3 Wound, were collected retrospectively. The patients had no other known comorbidities, such as diabetes, malnutrition, vascular disease, or immunodeficiency. After enzymatic debridement with NexoBrid, burn wounds were covered depending on the wound depth. Superficial partial-thickness burns were treated with Suprathel, this being the current standard of care in our unit. Deep partial-thickness burns were treated with either fish skin graft or an autologous STSG (0.2 mm, meshed 1:1.5) (see Figure 1). All the relative measurements were performed with the patients’ healthy skin as a control for the graft-treated areas.

### 2.2. Objective and Subjective Wound-Quality Assessment

The wounds of the burn patients were measured for their scar quality up to 12 months after injury. The after care of burn wounds following wound closure consisted of compression garments (Jobst^®^, Hamburg, Germany), daily application of moisturizing Panthenol, and sun protection with a solar factor ranging (SPF) from 30 to 50 for at least one year. For measurements of the stratum corneum’s hydration by electrical impedance, a Corneometer^®^ CM 825 (Courage + Khazaka, Cologne, Germany) was utilized, and a mean value was generated from five technical replicates. Similarly, the sebum content was measured by photometry with a Sebumeter^®^ SM 815 (Courage + Khazaka, Cologne, Germany). Skin elasticity was evaluated with a Cutometer^®^ (Courage + Khazaka, Cologne, Germany) based on the optical principle [22].

Furthermore, a simplified POSAS (Patient and Observer Scar Assessment Scale) was used to determine scar quality [23]. Therefore, the skin pliability, thickness, vascularity, pigmentation, and relief were the parameters evaluated by two independent assessors and served as the basis for the total observer scores. In addition, patients were asked about pain and itchiness using a visual analog scale. The scores for pain and itchiness were included in the total patient scores. For the total POSAS scores, all collected parameters were summed up (skin pliability, thickness, vascularity, pigmentation, relief, pain, and itchiness).

### 2.3. Wound-Size Measurement

The wound size was measured using Photoshops^®^ (Adobe Inc., San Jose, CA, USA). Anatomical landmarks were used for normalization, as described in a previous study to adjust for the influence of the variable distances of the photos [24]. Due to the initial enlargement of the wound area by split-thickness donor sites, the days until epithelialization were measured instead of the wound area.

### 2.4. Statistical Analysis

The results of the study are presented as the means ± standard errors of the mean (SEMs). *p*-values were calculated using the two-tailed unpaired *t*-test or Mann–Whitney *U* test when comparing two groups. When comparing more than two groups with unpaired data, ANOVA (analysis of variance) followed by a multiple-test comparison via Tukey’s post hoc test (homoscedasticity), or Brown–Forsythe or Welch ANOVA followed by Dunnett’s T3 post hoc test (heteroscedasticity) was performed. Statistical significance was considered at a *p*-value < 0.05.

## 3. Results

### 3.1. Patient Data

In total, 12 patients with dermal burns of different total burn surface areas (TBSAs) were treated with fish skin graft in our facility and included in the study. Seven deep partial-thickness burn wounds were covered with an STSG, eight superficial partial-thickness burn wounds were covered with Suprathel, and these were enrolled for comparison (see Table 1). The mean age of the patients was 41.8 ± 16.1, and the mean TBSA was 12.5 ± 9.4% (see Table 2). The cause of injury was thermal in all instances.

All the burns were subjected to enzymatic debridement on the second day after admission. The procedure included at least 12 h of soaking the wound with polyhexanide gel, 4 h of exposure to NexoBrid in an occlusive dressing, and 48 h of soaking with polyhexanide thereafter before applying the definitive therapy (fish skin graft, Suprathel, or an STSG). The procedure was a modified sequence, as described previously [25].

### 3.2. Application of Fish Skin Graft Reduced Time to Complete Healing Compared to Both Suprathel and STSGs

**Figure 2:**
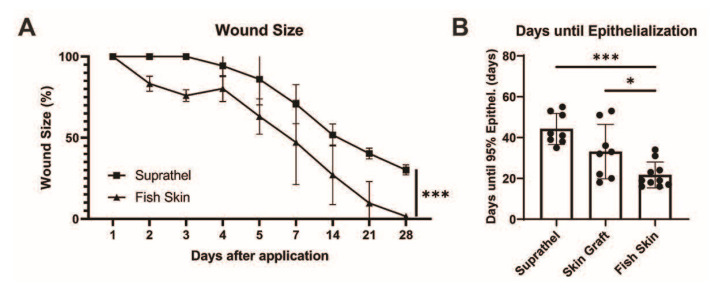

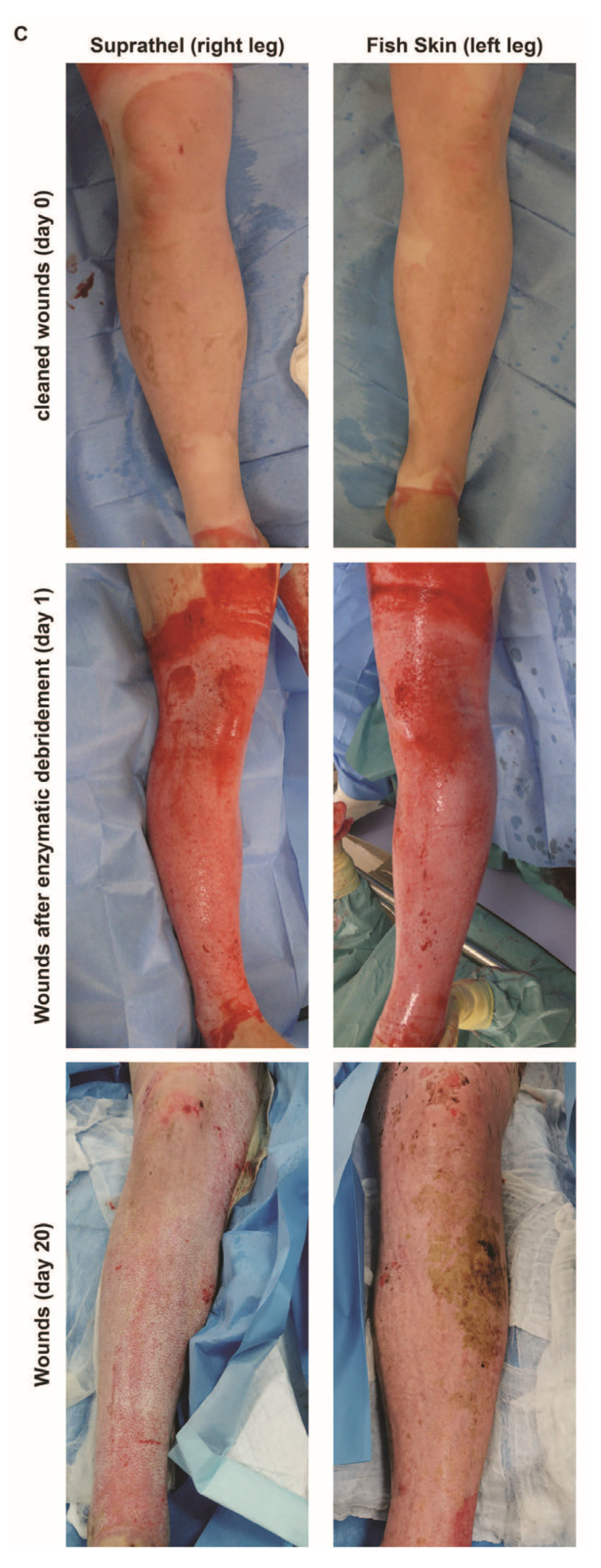
Total wound size over time. Based on daily and weekly measurements of the wound size by two independent assessors, the wound size was determined in Adobe Photoshop (Adobe Inc., USA). (**a**) There was a significant reduction in wound size over time in fish skin-treated wounds compared to Suprathel-treated wounds (*p* < 0.001). *n* = 12. Results are shown as the means ± SEMs. *p*-value: * < 0.05, ** < 0.01, and *** < 0.001; two-tailed unpaired *t*-test for pairwise analysis. ANOVA followed by multiple-test comparison via Tukey’s post hoc test (homoscedasticity) or Brown–Forsythe and Welch ANOVA followed by Dunnett’s T3 post hoc test (heteroscedasticity) for multi-group analysis. (**b**) Comparing the period from the application of the definitive wound closure to the point of 95% epithelialization: Suprathel (45.6 ± 6.6 days), STSG (34.7 ± 12.5 days), and fish skin graft (22 ± 6.3 days). (**c**) Examples of two deep dermal wounds in the same patient after enzymatic debridement treated with Suprathel (left image) and fish skin graft (right image). After 20 days, the Suprathel-covered wound showed almost no epithelialization, while the fish skin-treated wound had almost completely healed. As contralateral healthy skin was unavailable for the control, this case was not included in the measurements and is for demonstrative purposes only.

The wound surface area measured over 28 days after initial injury revealed accelerated wound healing in the fish skin-treated wounds compared to Suprathel-treated wounds, as shown by multiple-column *t*-tests (*p* < 0.001). There was also a significant difference in the area under the curve in Suprathel-treated (608.1 a.u.) and fish skin-treated wounds (437.4 a.u.) (*p* < 0.001) (see Figure 2a). Due to the lack of comparability of the STSGs in the wound-healing course, the days until epithelialization were measured instead of the wound area. The days until epithelialization were significantly lower in the fish skin-treated wounds (22 ± 6.3 days) than the Suprathel-treated (45.6 ± 6.6 days) and STSG-treated (34.7 ± 12.5 days) wounds (see Figure 2b).

### 3.3. Fish Skin Graft and STSG Resulted in Significantly Superior Elasticity in Regenerated Skin Compared to Suprathel

**Figure 3:**
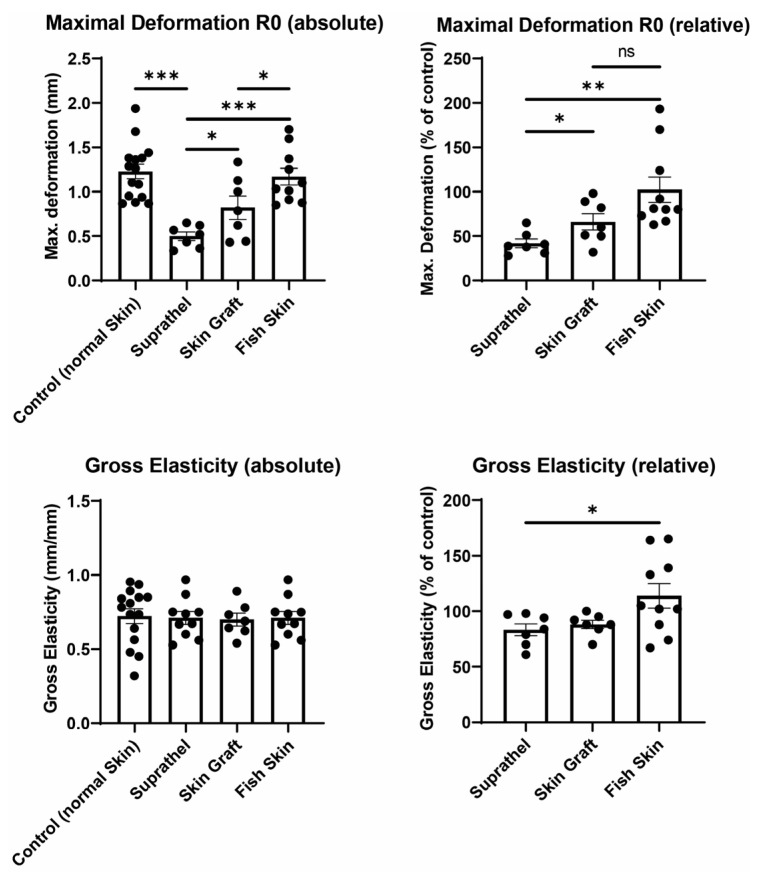
Elasticity of regenerated skin 12 months after injury. Using a Cutometer, skin deformation (R0) and gross elasticity (R2) were measured. Left graphs show an unpaired comparison of absolute values between all different wounds. Right graphs depict a paired comparison between wounds and healthy reference skin. Maximal relative deformation of wounds compared to healthy reference skin (top right): Suprathel (41.9% ± 4.8%), STSG (66% ± 9.1%), and fish skin graft (102.3% ± 14.3%). Relative gross elasticity of wounds compared to healthy reference skin (bottom right): Suprathel (83.3% ± 5.4%), STSG (88.1% ± 3.6%), and fish skin graft (114% ± 11%). *n* = 12. Results are shown as the means ± SEMs. *p*-value: * < 0.05, ** < 0.01, and *** < 0.001; two-tailed unpaired *t*-test for pairwise analysis. ANOVA followed by multiple-test comparison via Tukey’s post hoc test (homoscedasticity) or Brown–Forsythe and Welch ANOVA followed by Dunnett’s T3 post hoc test (heteroscedasticity) for multi-group analysis.

Next, we sought to determine the elasticity of the healed wound area (see Figure 3). The maximal deformation and gross elasticity were measured using a Cutometer 12 months after burn injury. The relative maximal deformation values (R0) after treatment with Suprathel (41.9% ± 4.8%), STSGs (66% ± 9.1%), and fish skin (102.3% ± 14.3%) were compared to those for normal skin. STSG- and fish skin-treated wounds showed significantly improved relative maximal deformation compared to Suprathel-treated wounds. The relative gross elasticity (R2) normalized to healthy skin was lower in Suprathel-treated wounds (83.3% ± 5.4%) compared to fish skin-treated wounds (114% ± 11%) 12 months after injury.

### 3.4. Fish Skin Graft Was Superior to Suprathel for Regenerated Skin’s Sebum Content and Resulted in Significantly Higher Water Content Than Both Suprathel and STSG

**Figure 4:**
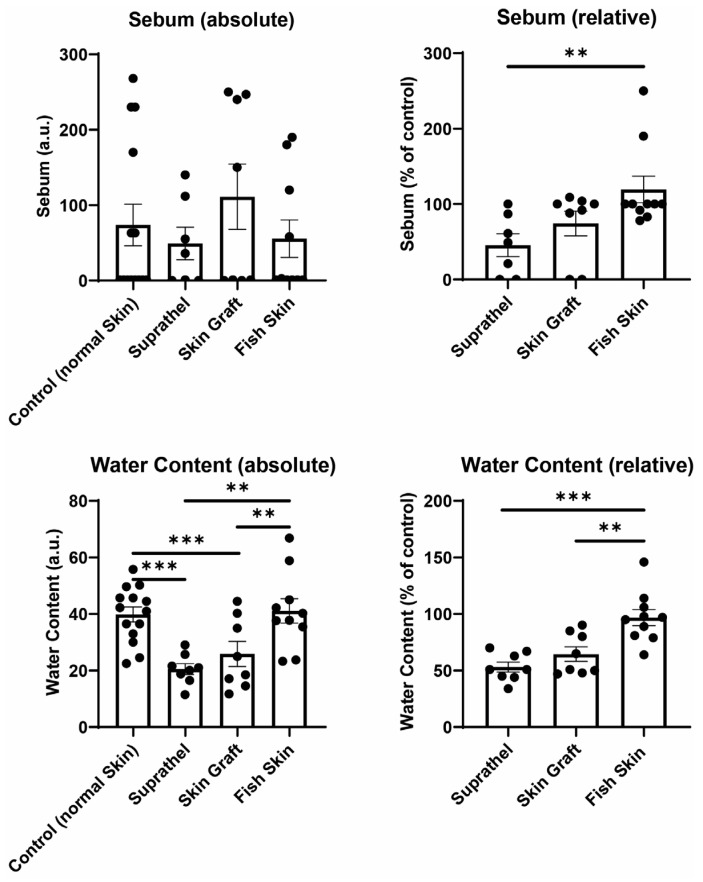
Sebum and water content of regenerated skin 12 months after injury. Using a Sebumeter and Corneometer, sebum and hydration of the stratum corneum were measured. Left graphs display an unpaired comparison of absolute numbers between all different wounds. Right graphs show a paired comparison between wounds and healthy reference skin. Relative sebum content of wounds compared to healthy reference skin (top right): Suprathel (45.4% ± 15.2%), STSG (74.1% ± 16.3%), and fish skin graft (119.3% ± 17.6%). The relative water content of wounds compared to healthy reference skin (bottom right): Suprathel (53.1% ± 4.4%), STSG (64.5% ± 6.4%), fish skin graft (96.9% ± 7.1%). *n* = 12. Results are shown as the means ± SEMs. *p*-value: * < 0.05, ** < 0.01, and *** < 0.001; Two-tailed unpaired *t*-test for pairwise analysis. ANOVA followed by multiple test comparison via Tukey’s post hoc test (homoscedasticity) or Brown–Forsythe and Welch ANOVA followed by Dunnett’s T3 post hoc test (heteroscedasticity) for multi-group analysis.

The sebum and water content of the healed wounds were investigated by utilizing a Sebumeter and Corneometer 12 months after burn injury (see Figure 4). The relative sebum and water content of skin areas treated with Suprathel, STSGs, and fish skin were compared to those of normal healthy skin. The fish skin-treated wounds exhibited significantly increased relative sebum content (119.3% ± 17.6%) compared to the Suprathel-treated wounds (45.4% ± 15.2%). Additionally, fish skin graft (119.3% ± 17.6%) tended to result in a higher relative sebum content compared to STSGs (74.1% ± 16.3%), but the difference was not significant. Regarding the relative water content, fish skin-treated wounds demonstrated a significantly higher relative water content (96.9% ± 7.1%) compared to Suprathel-treated (53.1% ± 4.4%) and STSG-treated wounds (64.5% ± 6.4%). No significant differences between the STSG- and Suprathel-treated groups were observed. The fish skin-treated wounds had a similar relative water content to healthy skin.

### 3.5. Fish Skin Graft Achieved Better Scar Quality Regarding Pliability, Thickness, Vascularity, Pigmentation, and Relief Categories as Well as Superior Alleviation of Pain and Itch Compared to Both Suprathel and STSGs

**Figure 5:**
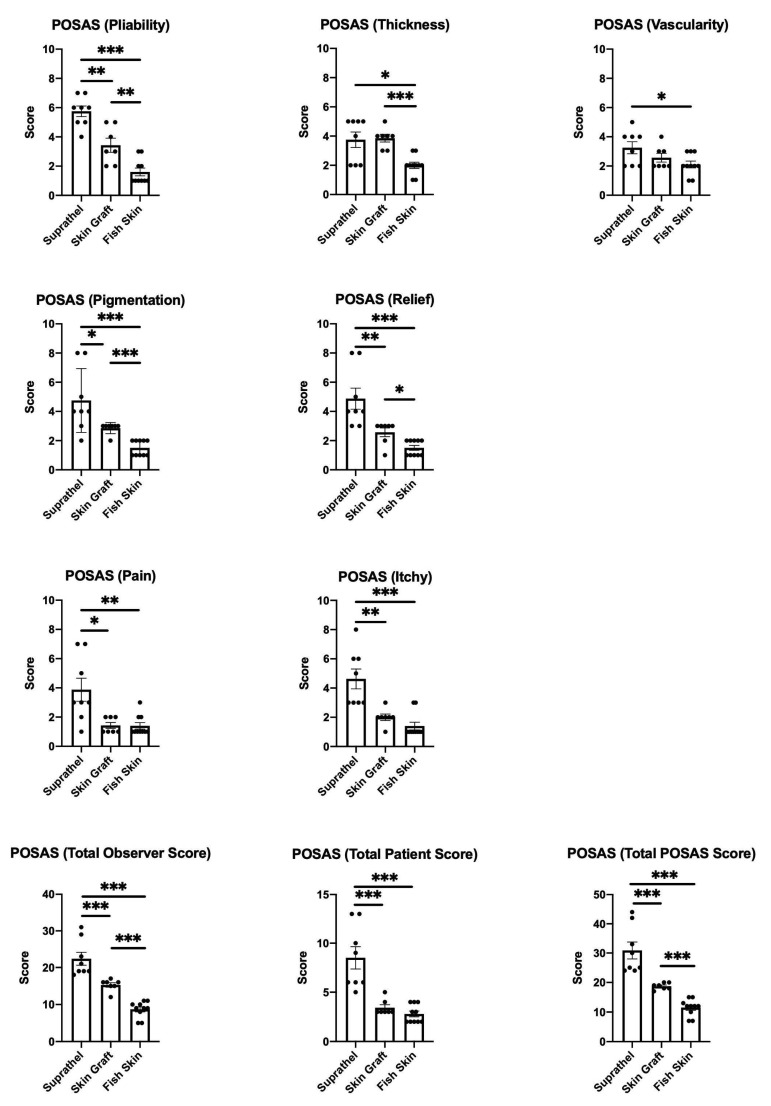
Patient and Observer Scar Assessment Scale (POSAS) 12 months after injury. Scoring of the pliability of the wounds was carried out by two independent assessors: Suprathel (5.75 ± 0.4), STSG (3.4 ± 0.5), and fish skin graft (1.6 ± 0.3). Scoring of the thickness of the wounds was carried out by two independent assessors: Suprathel (3.75 ± 0.5), STSG (3.9 ± 0.3), and fish skin graft (2 ± 0.2). Scoring of the vascularity of the wounds was carried out by two independent assessors: Suprathel (3.25 ± 0.4), STSG (2.6 ± 0.3), and fish skin graft (2.1 ± 0.2). Scoring of the pigmentation of the wounds was carried out by two independent assessors: Suprathel (4.75 ± 0.8), STSG (2.9 ± 0.1), and fish skin graft (1.5 ± 0.2). Scoring of the relief of the wounds was carried out by two independent assessors: Suprathel (4.9 ± 0.7), STSG (2.6 ± 0.3), and fish skin graft (1.5 ± 0.2). Scoring of patients’ pain involved surveying pain using a visual analog scale: Suprathel (3.9 ± 0.8), STSG (1.4 ± 0.2), and fish skin graft (1.4 ± 0.2). Scoring the itchiness of patients involved surveying itchiness using a visual analog scale: Suprathel (4.6 ± 0.7), STSG (2 ± 0.2), and fish skin graft (1.4 ± 0.3). The total observer scores for pliability, thickness, vascularity, pigmentation, and relief were summed up: Suprathel (22.38 ± 1.8), STSG (15.29 ± 0.6), and fish skin graft (8.7 ± 0.7). The total patient scores for pain and itchiness were summed up: Suprathel (8.5 ± 1.2), STSG (3.43 ± 0.3), and fish skin graft (2.8 ± 0.3). The total POSAS scores for pliability, thickness, vascularity, pigmentation, relief, pain, and itchiness were summed up: Suprathel (30.88 ± 2.9), STSG (18.71 ± 0.4), and fish skin graft (11.5 ± 0.9). *n* = 12. Results are shown as the means ± SEMs. *p*-value: * < 0.05, ** < 0.01, and *** < 0.001; Mann–Whitney *U* test for pairwise analysis. ANOVA followed by multiple-test comparison via Tukey’s post hoc test (homoscedasticity) or Brown–Forsythe and Welch ANOVA followed by Dunnett’s T3 post hoc test (heteroscedasticity) for multi-group analysis.

The POSAS scores were assessed 12 months after burn injury. The parameters of the POSAS scores in the Suprathel, STSG, and fish skin graft groups were compared (see Figure 5). The lower the score, the higher the similarity of the regenerated skin’s quality to that of normal skin. The fish skin treated group showed significantly lower scores for the pliability, thickness, vascularity, pigmentation, relief categories, and pain and itch scores than the Suprathel group. The fish skin treated group also showed significantly lower scores regarding the pliability, thickness, pigmentation, and relief parameters than the STSG group. The vascularity, pain, and itch scores were not significantly different between the skin graft and fish skin treated groups. Nevertheless, the fish skin treated group showed a trend for lower scores for vascularity and itch than the STSG group.

Furthermore, the STSG group was significantly superior to the Suprathel group in terms of pliability, pigmentation, relief, pain, and itch. The differences in thickness and vascularity between the STSG and Suprathel groups were not significant. The total patient scores were based on the scores for pain and itch, while the total observer scores were the sums of the scores for pliability, thickness, vascularity, pigmentation, and relief. The total POSAS scores were calculated based on all the aspects that were evaluated. For these, the fish skin graft and STSG groups showed significant superiority in all the scores compared to the Suprathel group. Furthermore, the fish skin graft group was significantly superior to the STSG group when comparing the total observer and total POSAS scores. The comparison of the total patient scores between the fish skin graft and STSG groups showed no significant differences.

## 4. Discussion

This study is an important step toward standardizing and optimizing burn care after enzymatic debridement. The results demonstrate a significant reduction in the healing time for fish skin-treated burn wounds compared to Suprathel-treated and STSG-treated wounds. The scar quality, characterized by the skin elasticity, hydration, and sebum content, in fish skin-treated wounds, was significantly better than that in the Suprathel group. Compared to that in the STSG group, the hydration was significantly higher in the fish skin treated group. Fish skin graft treatment tended to increase the skin elasticity and sebum content compared to STSG treatment, but the differences did not reach significance. In addition, fish skin graft resulted in significantly better POSAS scores than the Suprathel (pliability, thickness, vascularity, pigmentation, relief, pain, itch, total observer scores, total patient scores, and total POSAS scores) STSG groups (pliability, thickness, pigmentation, relief, total observer scores, and total POSAS scores).

The improvement seen in the wound healing process in the fish skin treated group reported herein is in line with the results of previous studies [2,13,14]. However, there are some limitations regarding the comparability of the three different substitutions. The indication of Suprathel differs based on the wound depth. While STSG is the current gold-standard treatment option for deep burn wounds, Suprathel is mainly used for superficial burns and represents the current standard of care for superficial burn wounds in our unit. In this study, fish skin graft was applied on mixed deep partial burn wounds comparable to the indication for STSG use. Therefore, for these indications, fish skin graft and STSGs are expected to be superior to Suprathel, as they achieve superior results by covering deeper burn wounds, which are more complicated to treat. However, the comparison of healing between Suprathel treatment for superficial, deep partial-thickness burns versus fish skin graft treatment for deeper burns is even more significant. Moreover, clinical assessment as a measurement for burn depth is limited by the subjectivity and clinical experience of the accessor [26]. Promising objective methods such as laser-doppler-imagination still face limitations and are not established yet [27]. Thus, clinical evaluation by burn specialists is still most commonly performed [27,28]. Furthermore, another complicating factor is the validation of wound healing in STSG wounds, as split-thickness donor sites cause an initial enlargement of the wound area due to wound creation at the donor sites. Therefore, in this study, the days until epithelialization were documented instead of the wound area in order to validate the wound-healing time for STSG wounds.

The results for scar quality are in accord with previous studies, which have reported good scar quality after fish skin graft coverage [4]. The Cutometer MPA 580, Sebumeter SM 815, and Corneometer CM 825 are the most utilized, objective, and valid methods used in research for skin-quality validation [29,30,31,32,33,34]. There are limitations in the comparability of absolute numbers. The skin condition and properties vary most notably by age and sex [35,36]. Therefore, we also generated relative numbers using the healthy reference skin of the same patient and defined an age range of 18 to 60 years. The comparison between absolute and relative measurements seemed to partially contradict the figures demonstrating the gross elasticity and sebum content. This was caused by the variability in the absolute numbers in the patients’ skin and the fact that we grouped control scores from all the burn wounds into a single control group. Being associated with lower scores in the control group, the fish skin treated group showed lower absolute values and, at the same time, a higher relative average sebum content compared to the STSG group. The same effect can be observed in the figures regarding gross elasticity. 

Although this is a small case series, the results did reach statistical significance. To ensure quality, the treatment of patients was standardized, excluding differences in the procedures of wound coverage. Nevertheless, further randomized controlled trials that include more cases are needed.

## 5. Conclusions

In conclusion, a combined treatment consisting of enzymatic debridement with NexoBrid, followed by coverage with an intact fish skin graft, was found to be superior to Suprathel and at least equal in some measures and superior in other outcomes to STSGs for deeper burns, while reducing the donor-site requirement with secondary wounds. Along with a reduction in wound-healing time, the advantages of fish skin graft treatment were better outcomes in the objective and subjective evaluation of scar quality. Therefore, a potential improvement in the quality of life of burn patients can be envisioned through the use of fish skin graft. Nevertheless, larger randomized controlled trials are needed to confirm these results.

## Figures and Tables

**Table 1:** This table shows the patients included in the study and each wound size with treatment.

Patient	TBSA (%)	Treated with Fish Skin Graft (%)	Treated with Suprathel (%)	Treated with Skin Graft (%)
**#1**	8	3	4	n.a.
**#2**	6	5	n.a.	1
**#3**	10	2	8	0
**#4**	1	0.5	n.a.	0.5
**#5**	5	3	2	n.a.
**#6**	20	1	5	14
**#7**	2	1	n.a.	1
**#8**	24	9	15	n.a.
**#9**	12	2	10	n.a.
**#10**	15	6	7	2
#11 *	35	17	n.a.	18
#12 *	12	2	10	n.a.

* Patients excluded due to unavailable contralateral control. n.a. = not applicable.

**Table 2:** Mean TBSAs of the patients and each group treated with fish skin graft, Suprathel, or STSGs.

**Patient**	**Total TBSA (%)**	**Treated with Fish Skin Graft (%)**	**Treated with Suprathel (%)**	**Treated with Skin Graft (%)**
Mean TBSA (%) *	12.5 ± 9.4	4.3 ± 4.5	7.6 ± 3.8	6.1 ± 7.1

* Mean TBSA including data of all 12 patients.

## Data Availability

Not applicable.
